# Correction: Laminin N-terminus α31 regulates corneal epithelial cell adhesion and migration through modifying the organization and proteolytic processing of laminin 332

**DOI:** 10.1371/journal.pone.0344484

**Published:** 2026-03-06

**Authors:** Lee D. Troughton, Valentina Iorio, Liam Shaw, Conor J. Sugden, Natasha D. Chavda, Peter Wilson, Djamilla Simoens, Andrea E. Conway, Stefano Sala, Simon Kaja, Kazuhiro Yamamoto, Kevin J. Hamill

The second author, Valentina Iorio, should be noted as having also contributed equally to this work.
