# Supplementary figures and images for: Laminin N-terminus α31 regulates corneal epithelial cell adhesion and migration through modifying the organization and proteolytic processing of laminin 332
Source: PLoS One. 2026 Jan 13;21(1):e0338797. doi: 10.1371/journal.pone.0338797 (PMC12798988; doi:10.1371/journal.pone.0338797)

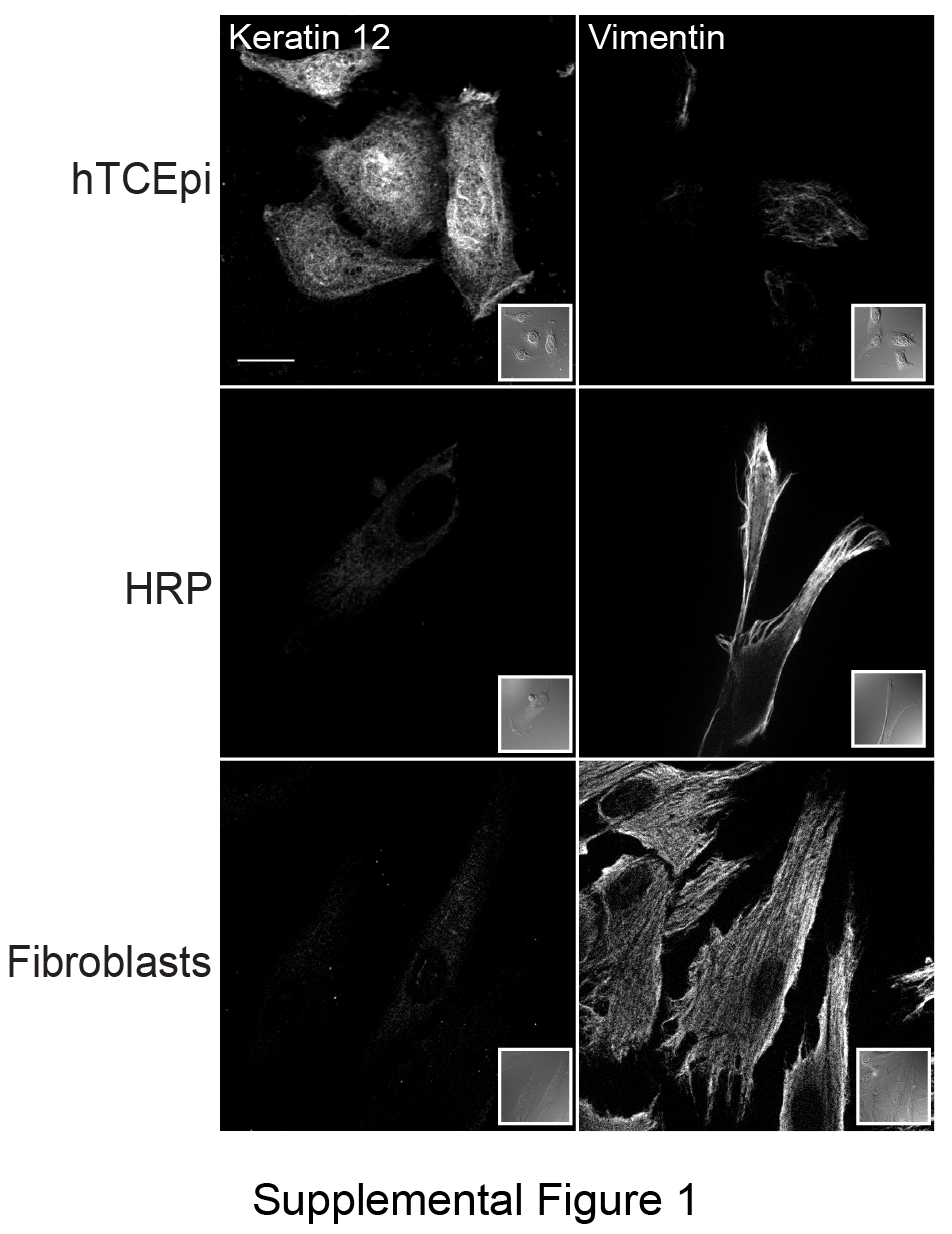

Supplement: S1 Fig — hTCEpi corneal epithelial cells, human retinal pericytes (HRP) or human dermal fibroblasts were plated overnight on glass coverslips then fixed and processed with antibodies against keratin 12 and vimentin. Scale bar 20 µm. (TIF) [file pone.0338797.s001.tif]

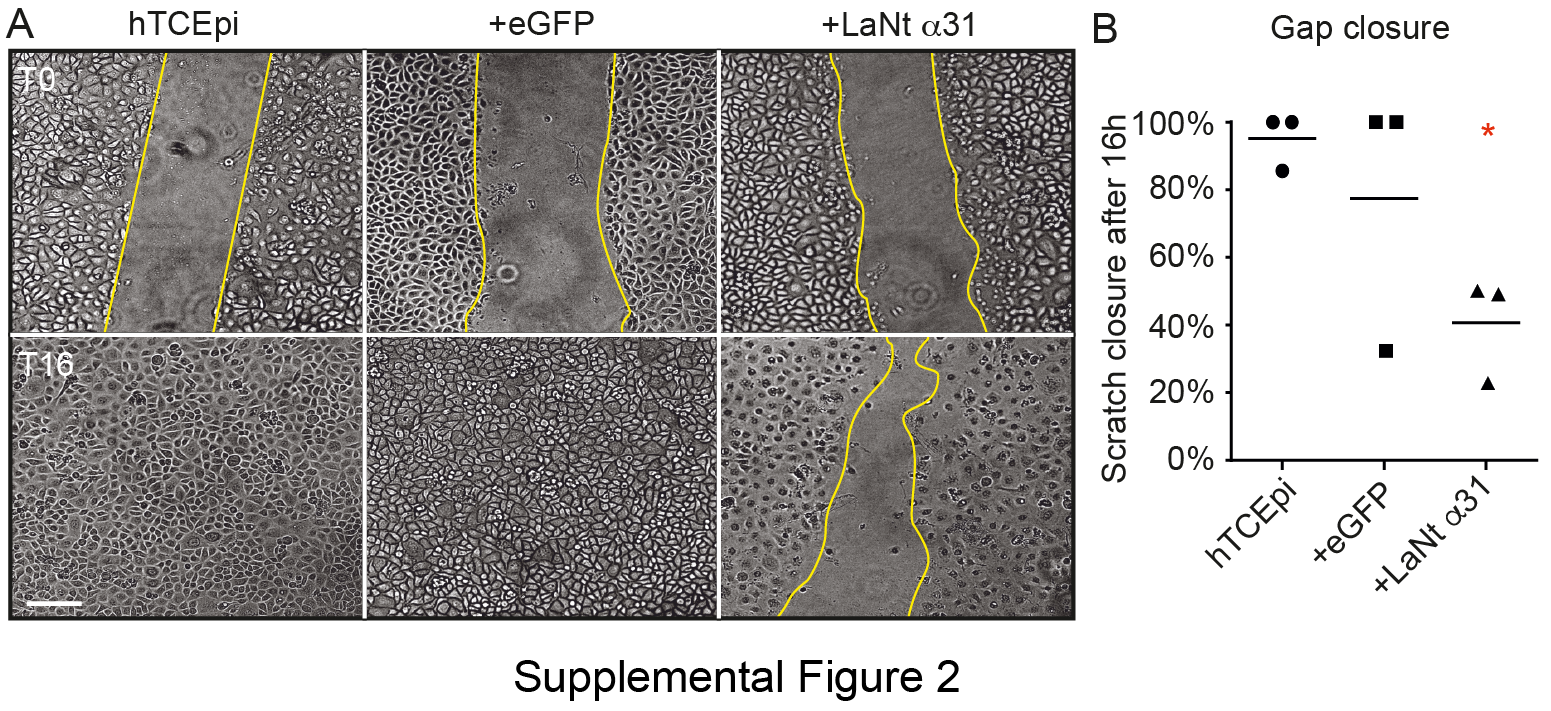

Supplement: S2 Fig — hTCEpi cells were plated at confluence ibidi® 2-well culture inserts, removing after 6 h. (A) Representative images from immediately after removing (T0 upper panels) and after 16 h of recovery (T16 lower panels), yellow lines indicate gap margins. Scale bar 100µm. (B) Gap area closure measured 16 h after removing inerts plotted as percentage of the initial gap area with each point representing an independent experiment with either 3 technical repeats per experiment. * denotes p < 0.05 compared with controls determined by one-way ANOVA followed by Bonferroni post hoc test. (TIF) [file pone.0338797.s002.tif]

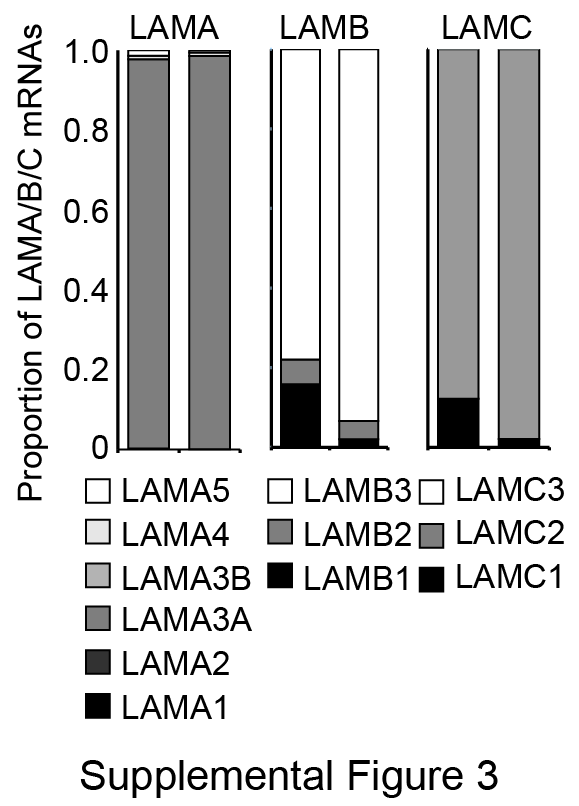

Supplement: S3 Fig — Total RNA was extracted from cells and RT-qPCR performed with primers specific to each laminin encoding gene. Bar chart represents the proportion of each isoform expressed, separated into LAMA, LAMB, and LAMC laminin isoforms. (TIF) [file pone.0338797.s003.tif]

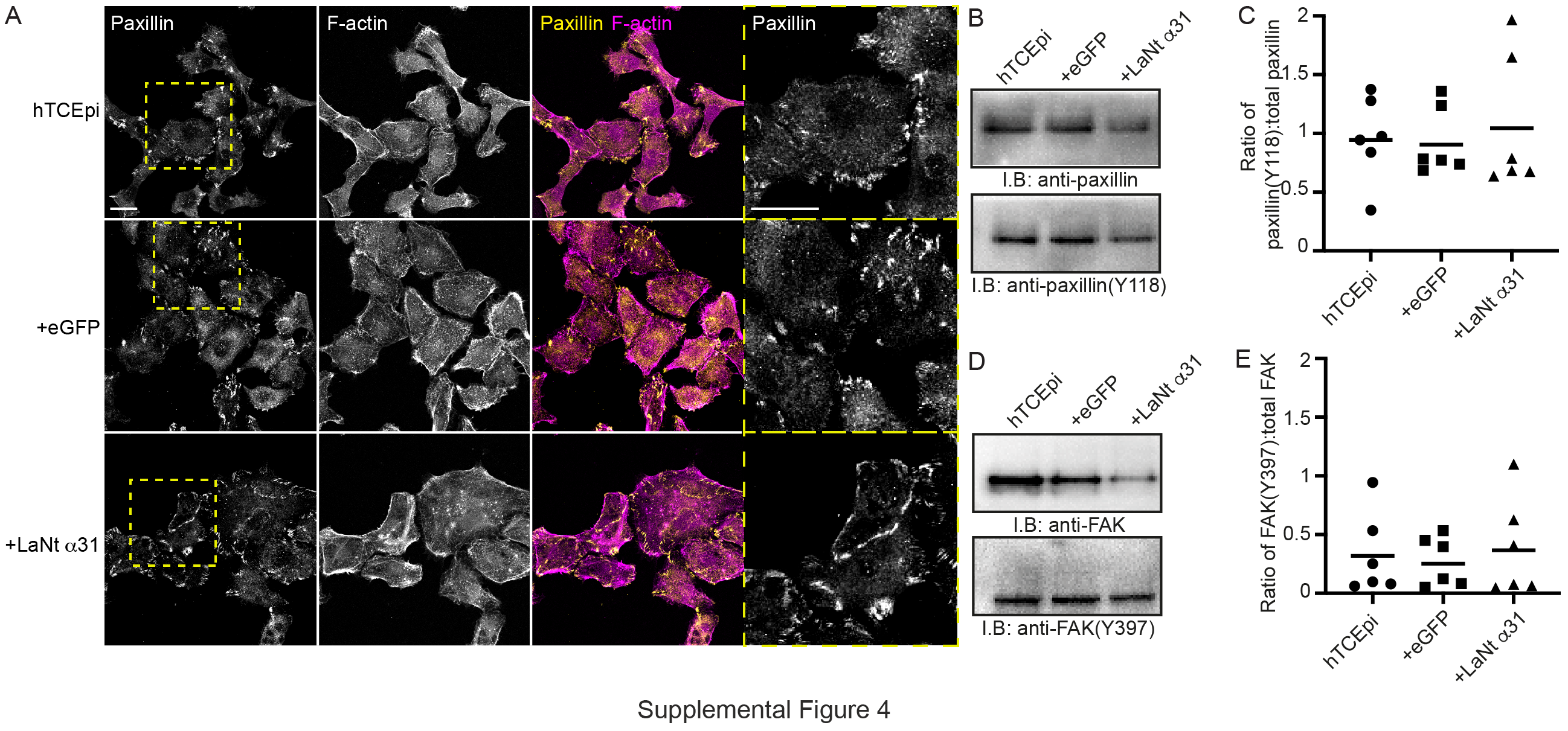

Supplement: S4 Fig — (A) Non-transduced, + eGFP, or +LaNt α31 hTCEpi were plated on glass coverslips then fixed and processed for indirect immunofluorescence with antibodies against paxillin and with phalloidin to label filamentous actin (F-actin). Scale bars 20 µm. (B) and (D) total protein extracts from the indicated cell treatments were immunoblotted with antibodies against paxillin and tyrosine 118 phosphorylated paxillin (Y118), or anti-focal adhesion kinase (FAK) or phosphorylated tyrosine 397 FAK (Y397). Dot plots in (C) and (E) are derived from densitometry analyses of immunoblots of the intensity of phosphorylated band/total protein. Each point represents an independent experiment, with lines indicating mean. Differences between groups did not reach statistical significance. (TIF) [file pone.0338797.s004.tif]

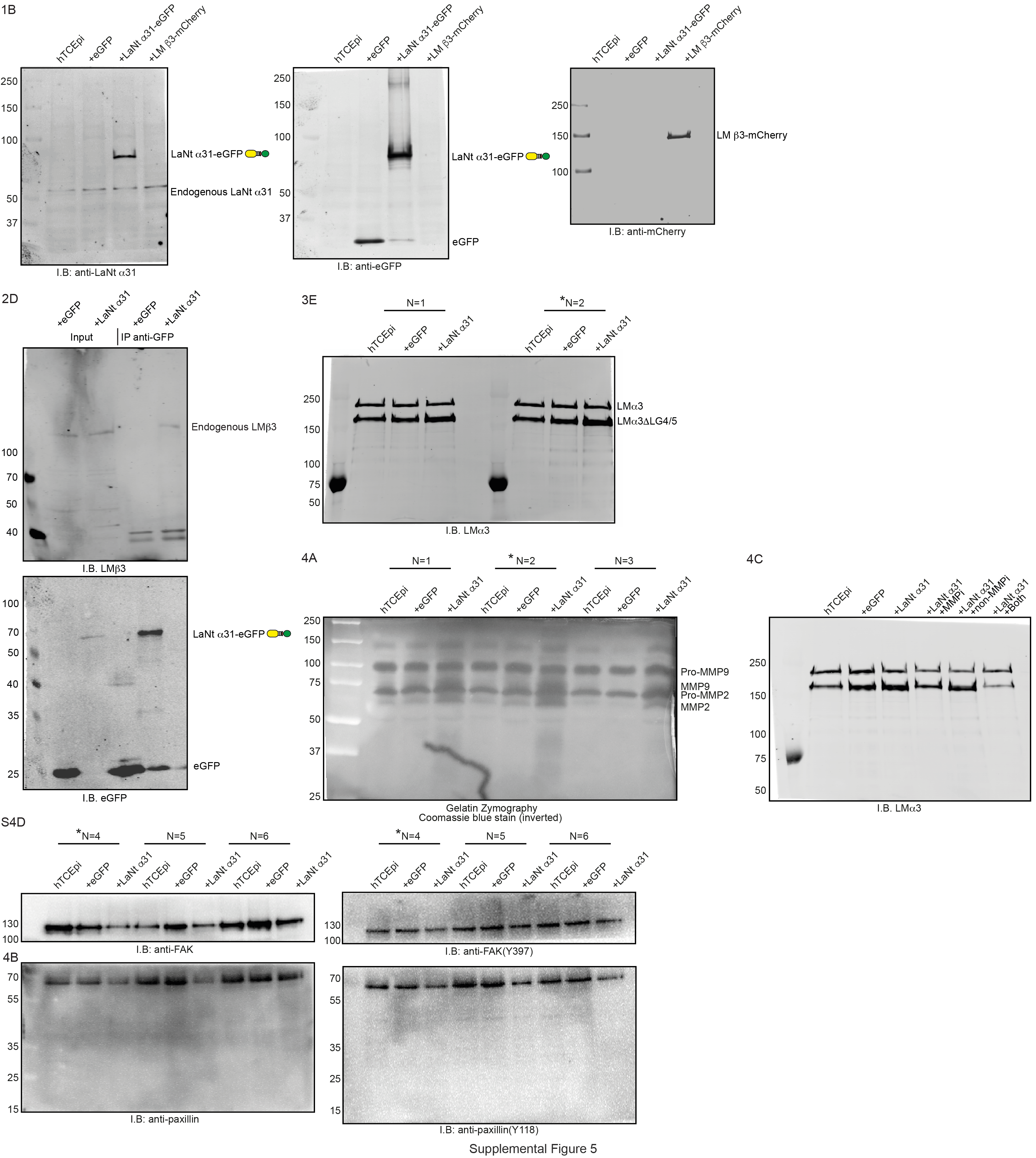

Supplement: S5 Fig — (TIF) [file pone.0338797.s005.tif]
